# Assessing a Digital Lifestyle Intervention to Reduce Dementia Risk in Older Adults

**DOI:** 10.1002/alz.094330

**Published:** 2025-01-09

**Authors:** Maria Bunyan, Jessica Irving, Jennifer H Barnett, Jamie Kawadler, Sarah Bauermeister, Philip Vassilev, John Gallacher, Ivan Koychev

**Affiliations:** ^1^ University of Oxford, Oxford, Oxon UK; ^2^ Five Lives, London, London UK; ^3^ University of Oxford, Oxford, Oxfordshire UK

## Abstract

**Background:**

An estimated ∼40% of dementia cases are due to modifiable risk factors which can be targeted in lifestyle interventions. Effective interventions employ face‐to‐face delivery, making them resource‐intensive and burdensome. Digital interventions offer scalability, accessibility and cost‐effectiveness. Engagement and efficacy of digital interventions are unknown, as is whether the ‘human component' of these interventions must be retained.

**Method:**

The Five Lives app is a digital health solution that combines a CE‐marked dementia risk assessment with a digital coaching programme (DC) to facilitate behaviour changes which could lead to eventual reduced dementia risk. Three groups were compared: DC‐only, DC and access to brief 1‐to‐1 virtual clinician services (DC+VC) and no intervention (control). The study spanned 12 weeks and participants (n = 154) were aged 50‐69 with normal cognition. A lifestyle score was conducted at baseline and exit, which scores participants on behaviour relating to sleep, physical activity, diet, stress & mood and mental stimulation. Engagement was measured as the total number of app activities (brain games, articles, quizzes and lifestyle log) completed during the intervention period.

**Result:**

Participant engagement was high (mean participant activities = 35/week). There was a significantly greater change in lifestyle score between intervention group (DC‐only and DC+VC pooled) and control (F(1,81) = 3.10, p = 0.049), and a trend for significance for a greater change in DC‐only versus control (F(1,24) = 3.96, p = 0.058). The change in lifestyle score between DC‐only and DC+VC was not significant, nor was the difference in app engagement. Total engagement was not predictive of lifestyle score change.

**Conclusion:**

Participant engagement indicates the intervention is feasible in older adults. Results suggest the Five Lives app is a promising tool to facilitate behaviour change to potentially reduce dementia risk, and the VC does not provide significant additional benefits. This study provides initial data to warrant further development of DC interventions to reduce dementia risk. Future analyses should investigate which participants respond best to the intervention, barriers to change, and how engagement interplays with sustained dementia‐targeted lifestyle change.

